# Out-of-pocket expenditures for primary health care in Tajikistan: a time-trend analysis

**DOI:** 10.1186/1472-6963-13-103

**Published:** 2013-03-18

**Authors:** Joëlle Schwarz, Kaspar Wyss, Zulfiya M Gulyamova, Soleh Sharipov

**Affiliations:** 1 Swiss Centre for International Health, Swiss Tropical and Public Health Institute, Socinstr 57, Basel, 4002, Switzerland; 2 University of Basel, Basel, Switzerland; 3 Project Sino, Rudaki prospekt proyezd 5, dom 1, Dushanbe, Tajikistan; 4 Centre of Sociological Researches “Zerkalo”, Loik Sharli str. 3, Dushanbe, Tajikistan

**Keywords:** Tajikistan, Informal payments, Out-of-pocket expenditure, Family medicine, Health reforms

## Abstract

**Background:**

Aligned with the international call for universal coverage of affordable and quality health care, the government of Tajikistan is undertaking reforms of its health system aiming amongst others at reducing the out-of-pocket expenditures (OPE) of patients seeking care. Household surveys were conducted in 2005, 2007, 2008 and 2011 to explore the scale and determinants of OPE of users in four district of Tajikistan, where health care is legally free of charge at the primary level.

**Methods:**

Using the data from four cross-sectional household surveys conducted between 2005 and 2011, time trends in OPE for consultation fees, drugs and transport costs of adult users of family medicine services were analysed. To investigate differences along the economic status, an asset index was constructed using principal component analysis.

**Results:**

Adjusted for inflation, OPE for primary care have substantially increased in the period 2005 to 2011. While the proportion of patients reporting the payment of informal consultation fees to providers and their amount were constant over time, the proportion of patients reporting expenditures for drugs has increased, and the median amounts have doubled from 5.3 US$ to 10.7 US$. Thus, the expenditures on medicine represent the biggest financial burden for patients accessing a primary care facility. Regression models showed that in 2011 patients from the most remote district with spread-out villages reported significant higher expenditures on medicine. Besides the steady increase in the median amount for OPE, the proportion of patients reporting making an informal payment to their care provider showed great variations across district of residence (between 20% and 73%) and economic status (between 33% among the ‘worst-off’ group and 68% among the ‘better-off’ group).

**Conclusions:**

In a context of limited governmental funds allocated to health and financing reforms aiming to improve financial access to primary care, the present paper indicates that in Tajikistan OPE – especially in relation to expenditures for drugs – have increased over time, and vary substantially across geographical areas and economic status. The fact that better-off households report disbursing more and in higher proportions hints towards a discrimination along the capacity to pay from providers. Increased public investments in the health sector, incentives for family doctors to provide PHC services free of charge and a strengthened drug control and supply system are necessary strategies to improve access of patients to services.

## Background

Currently there are strong international calls for the reform of health systems towards universal coverage and health financing schemes to ensure equity in access to care. In its 2010 World Health Report, the World Health Organisation (WHO) listed three fundamental barriers that hamper countries from reaching universal coverage: the limited availability of resources; an over-reliance on direct payments at the time people need care; and the inefficient and inequitable use of resources [1].

Direct payments by patients, or out-of-pocket expenditures (OPE), are a major obstacle in accessing health services and can lead to unexpected financial catastrophe, as such form of health financing does not allow a risk-pooling and cross-subsidisation based on solidarity between the sick and the healthy, the young and the elderly, the rich and the poor. In consequence, there is broad consensus on the importance that health care financing through household expenditures should be shifted to other sources such as governmental and/or health insurance.

As in other countries in Central Asia, household OPE for health are important in Tajikistan. According to WHO estimates, it is reported that around 67% of the total health expenditure in 2010 came from OPE [2]. According to the same estimates, this proportion of OPE in the total health expenditure has been decreasing since 2002 (when it peaked at 79%) as a result of the country’s economic growth and macroeconomic stability that has allowed increasing government investments in health as well as increasing international aid [2]. However, at 67%, the proportion of OPE in the total health expenditure is much higher in Tajikistan compared to its neighbouring countries such as Kyrgyzstan, Uzbekistan or Turkmenistan (respectively 38%, 43% and 41% in 2010) [2]. And overall, the total health expenditure, PPP per capita in 2010 in Tajikistan (128 US$) remains lower compared to the same neighbouring countries (respectively 140, 184 and 199 US$) [3]. As in other former soviet countries, state health workers in Tajikistan have very low wages, which results in informal payments and in-kind gifts constituting a complementary source of revenue for health professionals [4].

Though an important portion of household OPE occur at the secondary level due to the fact that a larger share of the overall services in Tajikistan is provided by specialists, the financing of primary care relies in an important way on patients as well [4]. Patients visiting a health centre may have to disburse cash or in-kind payments to different health professionals (i.e. doctor, nurse, lab worker) and for different services in or out of the facility (i.e. lab exams, transport, medicine). As Khodjamurodov and Rechel have reported, “although much of the discussion on out-of-pocket payments in Central Asia has focused on informal payments, expenditures on outpatient drugs, which always have been legal and required, might be more important in total magnitude and frequency than informal payments” [4]. In fact, expenditures on medicine have shown to be a large and increasing share of the total health expenditure of households [4]. Conducting a cross-sectional household survey in 2012 to analyse amongst others household expenditures for health seeking and identify the main barriers to the utilisation of mother and child health, birth assistance, vaccination and nutrition services in 18 districts of Tajikistan, it has been found that about two thirds of the patients’ expenditures were related to drugs [5]. Furthermore, conducting interviews with tuberculosis (TB) patients in Tajikistan, Ayé *et al.* collected information on medical and non-medical expenditures, as well as loss of income. Their study showed that the three largest shares of the direct costs of TB patients were special foods (29%), drugs (27%) and transport (25%), while medical fees expenditures represented only 7.3% of the direct costs [6]. The expenditures on drugs – legally free of charge for TB patients – concerned mainly additional, symptomatic treatment such as vitamin injections or intravenous rehydration, which are not essential drugs for TB treatment. In another context, Ridde *et al.* have similarly found in a mix-method study conducted in Burkina Faso on co-payment schemes for normal deliveries that women tend to pay more than the official fee. Three explanations are put forward: women had to pay for extra products that were not included in the delivery kit; practitioners had difficulty understanding the new policy; and unwillingness to apply the policy [7].

In 2007 the Government of Tajikistan introduced a Basic Benefit Package (BBP) scheme as part of its health reforms, to control for and reduce OPE amongst others. Though outlined as a national policy, the BBP was implemented and piloted in 8 districts, which included all 4 districts of observation of this study. The BBP regulates the entitlements of Tajik citizens to medical services, which at the primary health care (PHC) level are to be free of charge for all who live in the catchment area, except for certain laboratory and diagnostic tests. At the outpatient level, medicine is to be provided for free when prescribed by the family doctor for eight priority diseases [8]. In addition, the BBP aims at strengthening the role of family doctors as gate keepers of the health system by setting a lower co-payment fee at the secondary level for patients who have been formally referred by their family doctor. As some have observed, this policy is similar to reforms in Kyrgyzstan for instance, except that it did not include an accompanying institutional reform for the pooling of resources necessary to address the existent financial unbalances across health care levels and across districts due to a fragmented budget formulation [9,10]. The same have argued that despite a prosperous economic period since 2000 and increasing international aid investments, the lack of political stewardship has led to weak engagements in health reforms that have so far not shown to be sufficient instruments to address households financial burden in accessing health care [9,10]. Thus, though the proportion of OPE in the total health expenditure may have declined in the last years as an effect of increased government and foreign investments, this may not translate into a reduction of direct payments from patients in absolute figures. An internal evaluation of the Ministry of Health on the impact of the BBP conducted in 2008 at the hospital level comparing trends before and after the introduction of BBP in pilot and control districts has shown no reduction in the overall patient financial burden with utilising services, with the exception of deliveries and exempt patients [11].

In the context of Tajikistan, and under the BBP policy where the whole population is entitled to free primary care services, direct payments made to the family doctor can be considered as informal payments, defined as “direct contribution, which is made in addition to any contribution determined by the terms of entitlements, in cash or in kind, by patients or others acting on their behalf, to health care providers for services that the patients are entitled to” (Gaál *et al*. 2006, cited in [8]). Informal payments are not apprehended in this paper as a problem of corrupt practices, but as a symptom of the general condition of the Tajik health system that is under-financed and that lacks efficient and strong policy instruments [8].

To explore the scale and determinants of out-of-pocket expenditures of users of primary care services as well as time-trends, data collected through four cross sectional surveys in the period 2005 to 2011 were (re)analysed. The surveys were conducted within a bilateral aid project that aims at supporting the implementation of health reforms in pilot districts of Tajikistan, the Tajik-Swiss Health Sector Reform and Family Medicine Support Project (hereafter Project Sino). The period of observation coincides with a context of positive economic growth, population growth and inflation of the local currency which resulted in an overall improvement of the economic situation at the population level, expressed by the real GDP PPP$ per capita, except for a drop between 2005 and 2006 (Table 1).

**Table 1:** Macroeconomic indicators of Tajikistan covering the period of study

	**2005**	**2006**	**2007**	**2008**	**2009**	**2010**	**Mid-2011**
GDP growth (annual%)	6,7	7	7,8	8,9	3,8	6,5	6,9
Inflation rate (consumer price index)	7,1	10,0	13,1	20,5	6,4	6,4	7,7
Inflation adjustment factor	1,64	1,54	1,41	1,21	1,14	1,08	1,00
GDP per capita, PPP (current int.)	1500	1292	1598	1955	2030	2147	2236

Source: http://web.worldbank.org, and https://sdbs.adb.org/sdbs/jsp/index.jsp.

## Methods

### Study population and sampling strategy

The study population were PHC users living in rural districts of Tajikistan where Project Sino has been running it Family Medicine support activities: Dangara, Varzob, Shahrinav and Tursunzade. In 2005, only the first two districts were surveyed. Dangara district is situated South of Dushanbe and covers agricultural areas with spread-out villages and a population of approximately 107,000. Varzob is a more mountainous district North of Dushanbe characterised by remote areas with spread-out villages and a population of approx. 57,000. Shahrinav and Tursunzode are situated West of Dushanbe and are mostly agricultural plains with larger semi-urban centres, with a population of respectively 90,000 and 214,000. The latter two districts are economically better off compared to the former two.

A multi-level sampling strategy was used to select the study population. First, in the 2005 survey 15 PHC facilities were selected in Varzob and Dangara districts upon criteria of balancing remote and more accessible areas and representing 50% of the facilities in the two districts. In the following surveys, the exact same facilities were included, and 15 facilities were selected in Shahrinav and Tursunzade districts, respectively 6 and 9 facilities, in proportion with the number of facilities in each district and following the same criteria of balanced remote and more accessible areas. The second stage determined the selection of patients from each district proportionally to the number of adult patients who had, according to the national health information system, visited the facilities of each district in the previous year. Thus within each district the number of respondents from each facility was proportional to the total number of reported patients at that facility in the previous year. In 2005, 1000 patients were sampled as described in Dangara and Varzob. In 2007 and 2008, the targeted 2000 patients were first divided into two groups (1000 patients in Dangara and Varzob and 1000 patients in Shahrinav and Tursunzade) before applying the second stage sampling strategy. In 2011, the targeted 800 patients were first distributed in each district in proportions of 30% in both Dangara and Tursunzade (larger districts), and 20% in both Varzob and Shahrinav, to ensure representativity of each district. Then, the selection of patients by facility followed the same procedure as in the previous surveys. For each survey, adult patients (≥18 years) were selected for each health facility from the facility registrars of the previous month, starting with the most recent visitors of each facility, and running up the list selecting every third patient until the predefined number of respondents was reached (except in 2007 and 2008 when patients were selected randomly from the monthly list).

### Data collection

Patients were visited at home and surveyed with a questionnaire, identical in 2005, 2007 and 2008 and with small updates in 2011. Amongst others, the questionnaire included items to collect data on expenditure linked to the visit (payment or gift given to the family doctor, expenses on medicine and travel expenses to obtain the prescribed medicine) and general demographic information, including information on households’ assets. The questionnaire was administered by trained non-health professionals interviewers who were selected from the population of the districts. After three visits, if the person was not at home, the interviewer was to select the next patient up the facility registrar list. Oral consent was sought from the patient prior to starting the interview. The four surveys were conducted as part of the activities included in a jointly signed project agreement between the Ministry of Health of Tajikistan and the Swiss Agency for Development and Cooperation (which funds Project Sino). Ethical approval was provided for each survey by the Tajik Ministry of Health through a letter of authorisation for Project Sino to conduct the study. For three surveys (2005, 2007, 2008) data were collected amongst patients who had visited the PHC facility during the month of April and for one (2011) during the month of July.

### Data analysis

Data were analysed using Stata 11.2 software. For univariate analysis, Chi-squared test was used for binary and categorical variables and Kruskal-Wallis test to examine differences of medians (costs) between more than two groups. Multivariable regression models were used to explore the determinants of out-of-pocket expenditures. The models were constructed selecting independent variables based on univariate analysis and on evidence from the literature. Logistic regressions were run to calculate the odds ratios of binary variables such as the proportions of patients reporting giving money to the doctor, adjusting for the potential confounding effect of other variables (i.e. district and asset index). To control for the effect of non-normal distribution of numeric variables such as costs, the variables were transformed to log scale for the regression models, and for univariate analysis averages were presented using medians and means (transformed to log scale). To analyse the time trends in the expenditures of patients, the reported costs were adjusted to inflation using the consumer price index from the World Bank database [3], setting mid-2011 as a baseline (see Table 1), and then converted to US$ using mid-July 2011 foreign exchange rate (1 TJS = 0.214 US$).

An asset index was constructed for each survey based on collected information on asset ownership (telephone, car, fridge, etc.), characteristics of the household’s dwelling, water, sanitation and consumption of meat. While the 2005, 2007 and 2008 surveys collected the same asset variables, asset indicators were added to the 2011 survey based on the recent work of Ayé [12], allowing to account for change in households wealth related to economic growth (e.g. ownership of mobile phone). Principal Component Analysis (PCA) was used with each dataset to construct the asset index based on relative wealth ranking of the samples following Vyas and Kumaranayake’s guidelines [13]. For each survey, and following Filmer and Pritchett’s approach [14], the sample was stratified into three relative ‘economic groups’: the highest 20%, the middle 40% and the lowest 40%. For the construction of the 2011 asset index, some asset indicators were excluded from the PCA, as they showed either incoherent results (negative value of a positive asset), or an unequal distribution across districts, which reflects geographical differences in the distribution of an asset, rather than individual. For more information on the asset index construction, see Additional file 1.

## Results

### Characteristics of patients

Across the four surveys conducted in 2005, 2007, 2008 and 2011, final samples of respectively 889, 1935, 1768 and 787 adult patients interviewed on their experience with utilising primary health care facilities, and on the expenditures that their visit had implied were analysed. It was observed that the proportion of female respondents is generally higher and has increased over time, reflecting an increase in pregnancy-related visits. When excluding those pregnancy-related visits, the proportion of females is situated between 64 and 70% within the surveys and the median age ranks between 36 and 39 years (see Table 2). The median years of education for the total population scaled between 10 and 11 years, and the median number of reported visits in the last 12 months has increased from 2 visits in 2005 to 4 in 2011. As shown in Table 2, this increase in the number of reported visits to the PHC facility is not an effect of the increase in pregnancy-related visits.

**Table 2:** Characteristics of patients across the four surveys

	**2005**	**2007**	**2008**	**2011**
N	889	1935	1768	787
Female (%)	65.2	76.59	81.22	80.94
(without pregnancy-related visits)	(63.9)	(69.9)	(n/a)	(67.5)
Median age	37	33	31	28
(without pregnancy-related visits)	(38)	(36)	(n/a)	(39)
Median nb year of education	10	10	10	11
Median nb of visits in the last 12 months	2		3	4
(without pregancy-related visits)	(2)	3	(3)	(4)
Rayon				
Dangara (%)	71.8	26.2	28.4	29.7
Varzob (%)	28.2	22.3	23.6	19.6
Shahrinav (%)		11.8	12.8	20.3
Tursunzade (%)		39.7	35.2	30.4

### Total out-of-pocket expenditures

**Figure 1:**
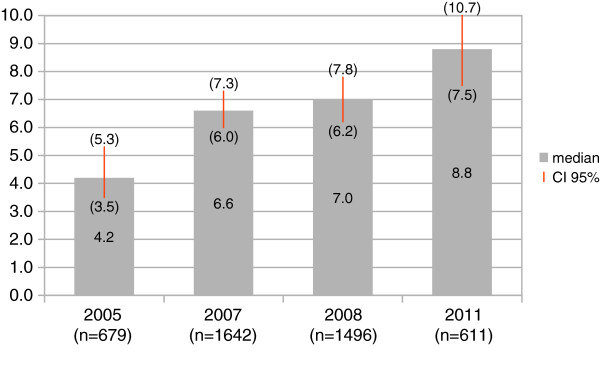
**Total median out-of-pocket expenditure, by year.** Kruskall-Wallis test for differences in median costs across years: χ^2^=65.24 (3df), P<0.001.

Figure 1 shows that between 2005 and 2011 the median total expenditure of the users of PHC facilities has more than doubled from 4.2 US$ to 8.8 US$. The median total expenditure is calculated cumulating the reported expenditures for prescribed medicine, travel costs to obtain the medicine and money given to the family doctor for all the respondents of the surveys, including zero values (i.e. patients who did not report any expenditures at all (14%)) and regardless of the reported prescription of medicine. The differences across the years are statistically significant at the 95% level (χ^2^=65.24(3df), P<0.001).

### Payments to the family doctor

Looking more specifically at the sources of expenditure shows that the reported increase is mainly due to the purchase of medicine rather than informal consultation fees. In fact, among patients who reported giving money to the family doctor (FD), the median amount given presented statistically significant but small non-linear differences across the surveys, ranking between 0.9 US$ (2007) and 1.3 US$ (2008), as shown in Table 3. Aside from this apparent stable informal consultation fee, the proportion of patients reporting giving money to the health care provider shows slight variations across the years, with higher proportions in 2008 (62%) and 2011 (49%). However, the logistic regression controlling for the effect of other factors showed that the odds of paying the FD were higher only in 2008, compared to 2005.

**Table 3:** Median and mean amount given to the FD, by year

	***Expenditure on medicine***			
	**2005**	**2007**	**2008**	**2011**
*N total in sample*	877	1935	1768	787
*% reporting a payment*^*a*^	45.6	44.1	61.6	49.3
*Median US$(2011)*	1.1	0.9	1.3	1.1
*Mean US$(2011)*	1.7	2.1	1.9	1.4
*(95% CI)*	(1.44 – 2.01)	(1.42 – 2.72)	(1.38 – 2.47)	(1.27 – 1.52)

^a^ % is the proportion of patients who reported giving money to the family doctor. χ^2^ test for differences in proportions across years: χ^2^=29.472(3df), P<0.001.

K-Wallis test of differences in median costs across years: χ^2^=29.472(3df), P<0.001.

The information on patients giving non-monetary gifts to the family doctor for the consultation was problematic, as many patients did not provide an estimated monetary value for the gift. In 2005, 15% of the patients reported making such in-kind gift of a value ranking between 0.4 and 14.3 US$. In 2007 and 2008, only 1.4% of the patients reported having given a gift to their FD. In 2011, over 21% reported making a gift, but only less than 1% of those provided a value for the gift. The proportion of patients reporting in-kind gifts was similar across the economic groups, and higher in Shahrinav and Tursunzade districts.

### Expenditures on medicine

The expenditures on medicine show a more pronounced trend. Indeed, the median amount spent on drugs has doubled in the six year span, from 5.3 US$ in 2005 to 10.7 US$ in 2011 (Table 4). The increase was not linear as 2007 patients have spent on average more on medicine compared to 2008. Moreover, the proportion of patients who were prescribed drugs by their family doctor has linearly increased from 77% to 84% over the same period. Positively, the proportion of patients who reported obtaining their prescribed medicine increased from 92% in 2005 to 98% in 2011.

**Table 4:** Median and mean expenditure on medicine, by year

	***Expenditure on medicine***			
	**2005**	**2007**	**2008**	**2011**
*N total in sample*	899	1935	1768	787
*% with prescription*	77.2	79.0	79.9	83.9
*% with expenditure*^a^	64.7	66.1	66.3	68.1
*Mean US$(2011)*	5.3	9.1	7.8	10.7
*Mean US$(2011)*	10.3	16.5	15.5	19.5
*(95% CI)*	(8.9 – 11.8)	(14.9 – 18.1)	(13.7 – 17.3)	(17.2 – 21.7)

^a^ % is the proportion of patients who reported expenditure on prescribed medicine. Kruskall-Wallis test of differences in median costs across years : χ^2^=117.34(3df), P<0.001.

Table 5 presents the median expenditure that patients spent travelling to obtain their prescribed medicine. The costs are small due to the fact that a lot of patients in the sample did not report any expenditures related to transport (40% in 2005, 37% in 2007, 41% in 2008 and 47% in 2011). However, when disaggregating the data by districts, travel costs appear to be unequally distributed in the samples, with patients from Varzob and Dangara tending to spend more on transport for drugs.

**Table 5:** Median and mean costs to travel for medicine, by year

	***Expenditure on medicine***			
	**2005**	**2007**	**2008**	**2011**
*N with prescription*	539	1372	1273	526
*% with reported costs*^*a*^	60.3	65.8	58.5	53.2
*Mean US$(2011)*	0.7	0.6	0.5	0.4
*Mean US$(2011)*	1.4	1.6	1.4	1.5
*(95% CI)*	(1.0 – 1.7)	(1.3 – 1.9)	(1.1 – 1.6)	(1.2 – 1.8)

ª % is the proportion of patients who reported travel costs to obtain medicine.

Kruskall-Wallis test of differences in median costs across years : χ^2^=12.179(3df), P=0.007.

### Determinants of out-of-pocket expenditures in 2011

As shown above and in the previous patient experience surveys and previous studies conducted in Tajikistan on households expenditure on health [15-18], there are differences in the mean expenditures of patients when testing for the influence of different factors, such as the district and the asset index. Exploring the determinants of out-of-pocket payments in 2011 using regression models showed that there were several factors having an effect on the expenditure of patients for medicine when controlling for the influence of other factor (Table 6): the district of residence, the reason to consult, the referral status, the self-assessed health status and the asset index. While some of these factors are expected to influence the expenditure for medicine, such as the self-assessed health status, the referral status and the number of visits, others are known as potential determinants of expenditure and signs of unequal access to care, such as the geographical area and the economic status of patients.

**Table 6:** Determinants of expenditure on medicine and of informal payments in 2011

		***Expenditure on medicine***			***Payment to the family doctor***		
***Characteristic***		***N***	***Coefficient (95% CI)***	***P-value***	***N***	***Coefficient (95% CI)***	***P-value***
Rayon							
	Dangara	*172*	1.00		47	1.00	
	Varzob	83	**2.18 (1.67, 2.86)**	**0.000**	*59*	**1.67 (1.40, 1.95)**	**0.000**
	Shahrinav	117	**1.72 (1.32, 2.20)**	**0.000**	*103*	**1.43 (1.22, 1.67)**	**0.000**
	Tursunzade	161	**1.43 (1.12, 1.84)**	**0.005**	169	**1.15 (1.00, 1.34)**	**0.052**
Reason to consult							
	diarrhoea	62	1.00		31	1.00	
	digestive (gastrointestinal)	33	1.25 (0.85, 1.82)	0.257	13	0.93 (0.71, 1.22)	0.606
	cardiovascular	34	1.40 (0.96, 2.08)	0.082	16	1.04 (0.78, 1.38)	0.795
	respiratory	47	1.14 (0.81, 1.62)	0.442	26	0.91 (0.74, 1.14)	0.435
	pregnancy	212	**0.76 (0.58, 1.00)**	**0.050**	231	0.96 (0.82, 1.14)	0.648
	genitourinary	36	1.30 (0.90, 1.88)	0.168	22	1.12 (0.89, 1.42)	0.334
	injuries	15	**0.58 (0.35, 0.96)**	**0.036**	6	1.32 (0.90, 1.93)	0.152
	anemia	19	**1.99 (1.25, 3.16)**	**0.004**	6	1.19 (0.82, 1.72)	0.369
	diabetes	26	1.21 (0.79, 1.88)	0.385	**5**	1.45 (0.97, 2.16)	0.067
	osteochondrosis	15	1.11 (0.65, 1.90)	0.699	2	0.98 (0.50, 1.90)	0.944
	other	34	**1.72 (1.16, 2.51)**	**0.006**	20	0.86 (0.68, 1.09)	0.215
Referral to specialist							
	not referred	248	1.00		156	1.00	
	referred	283	**1.63 (1.35, 1.95)**	**0.000**	221	1.05 (0.96, 1.15)	0.248
Self-assessed health status							
	very good	159	1.00		103	1.00	
	ok	328	**1.21 (1.01, 1.46)**	**0.042**	250	0.96 (0.87, 1.06)	0.481
	Poor / very poor	46	**1.97 (1.39, 2.80)**	**0.000**	25	1.08 (0.90, 1.32)	0.400
Economic position							
	Lowest 40%	214	1.00		101	1.00	
	Middle 40%	213	1.13 (0.94, 1.35)	0.201	175	1.09 (0.98, 1.22)	0.094
	Highest 20%	106	**1.32 (1.04, 1.70)**	**0.023**	102	**1.32 (1.17, 1.51)**	**0.000**

Coefficent: the mean of each category is compared to the baseline (1.00).

NB : only statistically significant determinants are presented in this table, and the significant determinants are in bold (P<0.05). CI : Confidence interval at 95% level.

Patients from Varzob, Shahrinav and Tursunzade who reported expenditures spent respectively 2.2, 1.7 and 1.4 times more on medicine compared to patients from Dangara district. The asset index appeared to be a determinant of the mean expenditure on medicine, with the group of 20% relatively ‘best-off’ respondents spending on average 1.3 times more compared to the 40% poorest group (baseline). The model also shows that the reason for which patients consulted PHC facilities presents also differences in mean expenditures on medicine when using diarrhoea as a baseline, with pregnancy and injury related consultations leading to lower expenditures, while anaemia and ‘other’ reasons increased the mean expenditures.

There were only two factors influencing the mean amount of money given to the family doctor for the sub-sample of patients who reported giving money in 2011 (48% of the patients): the district and the asset index (Table 6). Again, patients from Varzob, Shahrinav and Tursunzade districts paid on average more money to their doctor compared to Dangara patients. There were differences in the mean amount given to the doctor by economic groups, with the highest 20% economic group giving on average 1.3 times more money compared to the poorest 40% group.

Proportions of patients reporting making a payment to the family doctor showed interesting differences across districts and economic groups as well, as shown in Table 7. The odds of making an informal payment is between 7 to 8 times higher if the patient lives in one of the semi-urban district, adjusting for the effect of the economic status of patients; and the odds of making such payment is at least twice higher for the economically better-offs, adjusting for the effect of the district of residence.

**Table 7:** Proportion of patients reporting OPE in 2011, by district and economic status

		***N***	***Payment to doctor (%)ª***	***Prescription of medicine (%)***^***b***^
*District*				
	*Dangara*	234	20.1	84.2
	*Varzob*	154	40.9	76.6
	*Shahrinav*	160	65.0	91.3
	*Tursunzade*	239	72.8	83.3
*Economic position*				
	*Highest 20%*	156	68.0	83.3
	*Middle 40%*	316	56.7	82.6
	*Lowest 40%*	315	32.7	85.4

^a^ The differences are significant across districts (P<0.001) and across economic position (P<0.001).

^b^ The differences are significant across districts (P=0.006), but not across economic position.

## Discussion

The findings of this analysis show that the overall expenditures of primary health care patients in four study districts in Tajikistan, adjusted for inflation, have doubled between 2005 and 2011. This observation is mainly explained by an increase in the expenditures on medicine. In fact, not only do patients spend more money on prescribed drugs, but they are being prescribed and they obtain their medicine in larger proportions. The out-of-pocket expenditures were different across districts and across the relative economic groups with “better-off” patients reporting paying more (for drugs and for consultation) and in larger proportions.

Such results come in contrast with the climate of international momentum calling for affordable and quality health care for all and a reduction of the direct financial burden on the population accessing care. They also indicate that the health reforms being implemented in Tajikistan aiming to offer free primary care services, to reduce OPE and to strengthen the role of family medicine, namely the Basic Benefit Package, do not yield the expected benefits.

The period of observation however also coincides with a time of significant economic growth and increasing foreign aid, which has an impact on two aspects related to the results: the offer for health services and the demand for health care from patients. Through the reforms, the role of family doctors is being strengthened through training that aim at improving the diagnostic and treatment capacity, and thus the prescription of drugs for instance. Furthermore, the availability of drugs has been improving mainly through a growth of private retail points across the country. On the demand side, increasing purchasing power implies that Tajiks can allocate larger shares of their resources into health care. Though the surveys did not collect information on the revenues of patients, nor the capacity or willingness to pay for health care, the increase in the proportion of patients obtaining their prescribed drugs hints towards such phenomenon. Indeed, previous data from Tajikistan reported that the proportion of patients not being able to purchase their prescribed medicine was of 36% in 1999 [17], 9% in 2003 [15], 17% in 2005 [18], compared to the 2% in 2011.

The increase in both demand and supply sides are however not sufficient to ensure an equitable access to health care. Concerning the prescription of medicine, the findings have shown that there is an association between the amount of OPE spent on drugs and the relative economic position. The findings that ‘better-off’ patients pay higher prices for medicine may result from an access to better quality drugs or to trade-marks substituting for generic medicines, or from the fact that they are being prescribed drugs in larger amounts, as in the observations from Ayé *et al.* with TB patients being prescribed complementary drugs [6] and Ridde *et al.* with women paying for products not included in the basic delivery package [7]. Similarly, patients ranked higher economically pay their FD in higher proportions and in higher amounts, hinting towards a price discrimination mechanism but not necessarily unfair inequity in access to care. Indeed, the differences in median costs and proportions may reflect an informal cross-subsidisation system where health providers charge informal fees according to the capacity to pay of patients. The differences may however also reflect that wealthier patients pay their provider better and thus receive better care. In any case, such informal payment practice reflects in general the issue of an under-funded health system where health providers at the PHC level continue to have insufficient salaries and other inputs, and the gap is being filled through direct payments from patients.

Last, the disparities observed across districts also suggest that the improvements in the provision and utilisation of health services have been unequal across geographic areas. Patients from the most remote district (Varzob) paid more to obtain the medicine and encountered higher transport costs to obtain their medicine, thus suggesting issues of access to medicine for patients living in that district. Similarly, patients from the two semi-urban districts reported giving money to the FD in significantly higher proportions and in higher amounts. Though such disparities were expected as the districts of observation are economically unequal, the findings raise questions on the allocation of resources across regions of the country and subsequent issues of access to care and the quality of care received by patients in certain districts.

### Limitations

The set of four cross-sectional studies show some sampling differences, for instance the increasing share of female respondents. As the increase of women may have been due to the effect of safe motherhood campaigns efforts, costs analyses were re-run excluding all the pregnancy-related visits in the 2005, 2007 and 2011 datasets and the findings did not show differences in the median costs when compared to the analyses with the entire sample. The over-representation of females in general across all four surveys-remaining present when excluding the pregnancy-related consultations - could be attributed to a selection bias, as interviews were conducted at home during the day, when men are most likely to be working outside of the household. Such men would have been replaced by the next patient on the list after three unsuccessful visits, thus leading to their under-representation. This bias appears not to be problematic for the analyses, as in the regression models assessing the determinants of expenditures, sex was not a factor significantly influencing the models (coeff.= 0.9, P=0.42 for costs of medicine and coeff.=0.88, P=0.15 for payments to FD). Thus, it was decided not to weight the datasets to take into account the increasing proportion of females.

The ‘reason to consult’ information is to be interpreted with caution, as the variable does not allow to distinguish preventive from curative care related visits. The difference in the season for the collection of data did not show differences in the trends and distribution of diseases. Recall bias from the respondents on expenditures was minimised by the fact that patients had visited their health facility at the maximum two month before the interview, and the courtesy bias linked to the question on informal payments to the doctor was minimised by the fact that interviews were conducted at home and by non-health professionals. Also, patients may not have been reluctant in reporting informal payments because OPE have been widespread practice since the communist period in Central Asia [8]. The information on in-kind gifts given to FD was weak as patients did not provide a monetary value for it, especially in 2011, suggesting that the reported informal payments to FD may have been minimised in the survey.

Finally, the discussion on the two-fold increase in expenditures on drugs is limited by the fact that the data does not include information on the clinical appropriateness of the prescription by the physician, on how and where the drugs were obtained, nor on the type of medicines obtained by patients (generic medicine or trade-marks). And though it is not possible with the available data to know which patients fall under the BBP regulation that entitles them to free drugs (there are eight types of priority diseases which entitle patients to free medicine), it can however be stated that such reform on access to drugs aims at reducing the expenditure of patients on medicine overall.

## Conclusions

The time series analysis of four surveys conducted between 2005 and 2011 in rural Tajikistan shows that across the time-span the practice of informal payments has not decreased, and out-of-pocket expenditures on medicine has doubled. This observation comes in contradiction with the national efforts to strengthen the current health financing reforms towards equitable and affordable access to primary health care services and international calls for universal coverage. In 2011, there were differences across the geographical regions of Tajikistan in out-of-pocket expenditures, as well as across the relative economic groups. The fact that ‘better-off’ patients report paying substantially more and in higher proportions hints towards a discrimination from providers along the capacity to pay of patients.

Thus, it can be concluded that if the main aim of the health reforms is to improve the performance of the health system in offering affordable and equitable health care services, the financial barriers that patients face when utilising PHC must be addressed. In fact, the BBP policy alone proves not to be a sufficient instrument if not accompanied by parallel efforts to improve delivery of PHC services such as incentives for family doctors to deliver quality care, improved prescription rationality, and measures to guarantee an affordable and equitable drug supply system. Such measures require increased public investments in the health sector, as seen in neighbouring countries.

## Competing interests

The authors declare that they have no competing interests.

## Authors’ contributions

JS designed the 2011 survey, supervised data collection in 2011, conducted the re-analysis on all four surveys and led the writing up of the manuscript. SS conducted the 2007 and 2008 surveys (data collection and analysis), and organised data collection and entry in 2011. ZG contributed with the outline of the study, design of the questionnaire and in the data collection. KW contributed to the conception of the four surveys and in the writing up of the manuscript. All authors read and approved the final manuscript.

## Pre-publication history

The pre-publication history for this paper can be accessed here:

http://www.biomedcentral.com/1472-6963/13/103/prepub

## Supplementary Material

Additional file 1 Appendix Construction of the asset index. Click here for file

## References

[B1] World Health Organization Health systems financing: the path to universal coverage 2010 10.2471/BLT.10.078741 PMC2878164 20539847

[B2] European health for all database (HFA-DB) http://data.euro.who.int/hfadb/

[B3] World data bankhttp://databank.worldbank.org/ddp/home.do

[B4] Khodjamurodov G Rechel B Health systems in transition Health Syst Transit 2010 12 v–xix 1–154 21132994

[B5] Steinmann P Kiefer S Kamoliddinov P Matthys B Hanlon P Wyss K Demand and supply-side incentives to increase utilization of primary health care services in Tajikistan 2012 The World Bank, Rapid Social Response, Zerkalo: Swiss Tropical and Public Health Institute

[B6] Ayé R Wyss K Abdualimova H Saidaliev S Household costs of illness during different phases of tuberculosis treatment in Central Asia: a patient survey in Tajikistan BMC Publ Health 2010 10 18 10.1186/1471-2458-10-18 PMC2822824 20078897

[B7] Ridde V Kouanda S Yameogo M Kadio K Bado A Why do women pay more than they should? a mixed methods study of the implementation gap in a policy to subsidize the costs of deliveries in Burkina Faso Eval Program Plann 2013 36 145 152 10.1016/j.evalprogplan.2012.09.005 23123308

[B8] Gaál P Jakab M Shishkin S Kutzin J Cashin C Jakab M Strategies to address informal payments for health care Implementing health financing reform: lessons from countries in transition 2010 Copenhagen, Denmark: European Observatory on Health Systems and Policies 327 363

[B9] Rechel B Khodjamurodov G International involvement and national health governance: the basic benefit package in Tajikistan Soc Sci Med 2010 70 1928 1932 10.1016/j.socscimed.2010.02.029 20363064

[B10] Rechel B Ahmedov M Akkazieva B Katsaga A Khodjamurodov G McKee M Lessons from two decades of health reform in Central Asia Health Policy Plan 2012 27 4 281 287 10.1093/heapol/czr040 21609971

[B11] Jakab M Akkazieva B Kojokeev K Manjieva E Bobohodjaeva Z Magzumova F The basic benefit package and patient financial burden at the hospital leve. An Intermediate Investigation 2008 Report from the World Health Organisation Europe and the Ministry of Health of the Republic of Tajikistan

[B12] Ayé R Determinants of household costs and access to care for tuberculosis in Tajikistan. PhD Dissertation 2010 University of Basel: Faculty of Science

[B13] Vyas S Kumaranayake L Constructing socio-economic status indices: how to use principal components analysis Health Policy Plan 2006 21 459 468 10.1093/heapol/czl029 17030551

[B14] Filmer D Pritchett L Estimating wealth effects without expenditure data–or tears: an application to educational enrollments in states of India Demography 2001 38 115 132 11227840 10.1353/dem.2001.0003

[B15] Habibov N Determinants of out-of-pocket expenditures on prescribed medications in Tajikistan: implications for healthcare sector reform J Health Organ Manag 2009 23 170 182 10.1108/14777260910960911 19711776

[B16] Habibov NN The inequity in out-of-pocket expenditures for healthcare in Tajikistan: evidence and implications from a nationally representative survey Int J Public Health 2010 56 397 406 20862599 10.1007/s00038-010-0193-9

[B17] Falkingham J Poverty, out-of-pocket payments and access to health care: evidence from Tajikistan Soc Sci Med 2004 58 247 258 10.1016/S0277-9536(03)00008-X 14604611

[B18] Tediosi F Aye R Ibodova S Thompson R Wyss K Access to medicines and out of pocket payments for primary care: evidence from family medicine users in rural Tajikistan BMC Health Serv Res 2008 8 109 10.1186/1472-6963-8-109 18500995 PMC2413230

