# Supplementary material for: Out-of-pocket expenditures for primary health care in Tajikistan: a time-trend analysis
Source: BMC Health Serv Res. 2013 Mar 18;13:103. doi: 10.1186/1472-6963-13-103 (PMC3614449; doi:10.1186/1472-6963-13-103)
Supplement: Additional file 1 — Appendix Construction of the asset index. [file 1472-6963-13-103-S1.pdf]

Appendix: Construction of the asset index

Table 1 shows the indicators used for the asset index in the 2005, 2007 and 2008 PCAs, and the coefficients attributed to each indicator each year.

For the 2011 survey, the following indicators were collected: crowding, sources of drinking water (river; well, public tap, tap at home or delivered), type of toilet, type of floor in the house, type of walls in the house, meat consumption frequency, bike, motorcycle, car, donkey, cow, sheep, goat, land, fridge, video, dvd, land phone, mobile phone.

When plotting all collected variables into the PCA in Stata software, the following variables were assigned incoherent signs: ownership of a car, of a motorbike, of a bicycle, of animals (all animals) and of land. They were all given negative coefficients, where they should have been positive. To control for the potential effect of geographical differences causing the incoherent sign attribution, two different asset indices were constructed using PCA, one for Varzob and Dangara, more remote and rural areas, and one for Tursunzade and Shakhinav, slightly better-off. The two PCAs showed unsatisfactory results as well. Cattle and land assets became positive in the PCA for Dangara and Varzob as expected, but other asset variables turned out to be weighted incoherently (crowding was a positive asset, tap at home a negative one). In the PCA for Shakhinav and Tursunzade, all the households characteristics had factors that had the opposite sign from expected (positive crowding, water from river, negative flush toilet, fridge, tv, etc.). Thus, the strategy of running two PCAs to construct one combined asset index was abandoned. Also, as the weights attributed to cattle and land asset variables were relatively small (-0.12 to -0.23), and as the proportion of unexplained variance was relatively high (0.82 to 0.94), it was decided not to include those variables in the final PCA. Once the animals and land ownership variables were taken out, the PCA attributed coherent signs to the ownership of transport means (the inverse manipulation was not the case). It was thus decided to keep the latter included.

Once the final asset index constructed, each variable was cross-tabulated with the economic groups created, to check for distribution. Based on this bivariate analysis, three more variables were finally excluded from the asset index: well as a source of drinking water, dwellings of the house, and land phone, as they reflected geographical differences rather than individual in their distribution.

The final PCA model run for the 2011 survey is as shown in Table 2.

Tables

Table 1: Asset index constructed for 2005, 2007 and 2008 datasets

2005 Variable	Coeff.	2007 Variable	Coeff.	2008 Variable	Coeff.
crowding	-0.03	crowding	-0.14	crowding	-0.08
water : tap at home	0.26	toilet : flush toilet	0.65	water : tap at home	0.51
water : public tap	-0.27	meat consumption freq.	0.35	water : public tap	-0.06
toilet : hole	0.63	car	0.08	water : hand pump	-0.08
no toilet	-0.59	tv	0.04	water : electrical pump	-0.06
meat consumption freq.	0.17	dwellings of the house	0.66	water : river	-0.21
car	0.22			water : spring	-0.16
tv	0.05			water : delivered at home	-0.15
dwellings of the house	0.2			water : well	-0.03
				water : ditch	-0.14
				toilet : flush toilet	0.54
				meat consumption freq.	0.09
				car	0.02
				tv	0.04
				dwelling of the house	0.55
Component 1 Eigenvalue	1.82		1.61		2.04

Table 2: asset index constructed for the 2011 dataset

Variables*	Coeff	Unexplained
crowding	-0.11	0.97
water : river	-0.20	0.90
water : public tap	-0.32	0.75
water : tap at home or delivered	0.48	0.45
flush toilet	0.44	0.54
meat consumption freq.	0.20	0.91
bike	0.02	0.99
moto	0.07	0.99
car	0.11	0.97
fridge	0.46	0.48
tv	0.14	0.95
video	0.18	0.93
dvd	0.21	0.89
mobile phone	0.24	0.86
Eigenvalue of Comp(1)	2.40	

* all variables were binary, except for crowding, meat consumption and Mobile phone ownerships, which were either count or catgorical.
